# Supplementary material for: Association between oral microbiome and seven types of cancers in East Asian population: a two-sample Mendelian randomization analysis
Source: Front Mol Biosci. 2023 Nov 21;10:1327893. doi: 10.3389/fmolb.2023.1327893 (PMC10702768; doi:10.3389/fmolb.2023.1327893)
Supplement: Supplementary file 1 [file DataSheet1.ZIP › Supplementary Materials/MR plots for tongue/tongue═╝/Ovarian cancer/pheno.1073_to_ovarian cancer_leave_one_out.pdf]

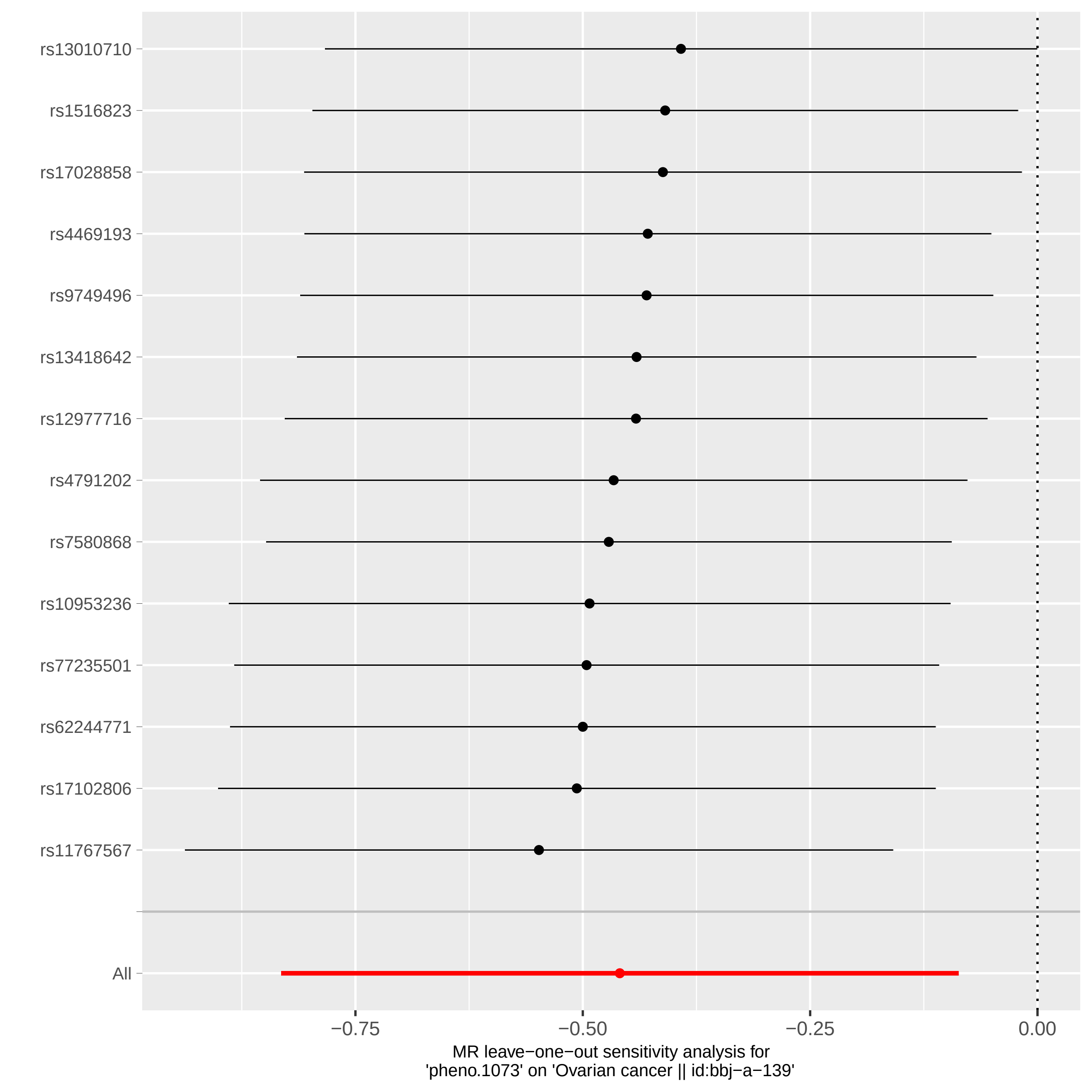
rs13010710
rs1516823
rs17028858
rs4469193
rs9749496
rs13418642
rs12977716
rs4791202
rs7580868
rs10953236
rs77235501
rs62244771
rs17102806
rs11767567
All
−0.75
−0.50
−0.25
0.00
MR leave−one−out sensitivity analysis for
'pheno.1073' on 'Ovarian cancer || id:bbj−a−139'
